# A new bacterial blight resistance gene *Xa50*(*t*) in the *Xa4* locus confers resistance against *Xanthomonas oryzae* pv. *oryzae* in rice

**DOI:** 10.3389/fpls.2025.1657476

**Published:** 2025-08-29

**Authors:** Man Li, Muhammad Sohaib Shafique, Houyu Zhou, Jialu Wang, Yapei Liu, Chunlian Wang, Zhonghua Wang, Zhiyuan Ji

**Affiliations:** ^1^ State Key Laboratory of Crop Stress Resistance and High-Efficiency Production, College of Agronomy, Northwest A&F University, Yangling, Shaanxi, China; ^2^ State Key Laboratory of Crop Gene Resources and Breeding/National Key Facility for Crop Gene Resources and Genetic Improvement, Institute of Crop Sciences, Chinese Academy of Agricultural Sciences, Beijing, China

**Keywords:** bacterial blight resistance, Xa50(t), *Xanthomonas oryzae* pv. oryzae (Xoo), wall-associated kinase (WAK), Xa4

## Abstract

Rice bacterial leaf blight (BB), caused by *Xanthomonas oryzae* pv. *oryzae* (*Xoo*), leads to severe yield losses in rice. Resistance breeding is a sustainable approach to mitigate the impact of this disease. In this study, a novel BB resistance gene, *Xa50*(*t*), was identified in the germplasm line CX315. Genetic analysis revealed that the resistance is conferred by a dominant resistant gene, tentatively named *Xa50*(*t*), which provides broad-spectrum and robust resistance to multiple *Xoo* strains. Using an F_2_ population derived from a cross between CX315 and IR24, *Xa50*(*t*) was fine-mapped to a 147.7 kb region on the long arm of chromosome 11 flanked by InDel markers M11–588 and M11-602. Gene expression analysis identified three candidate genes out of 13 open reading frames (ORFs) predicted in candidate region. *ORF5* and *ORF9* (*Xa4*), encoding a wall-associated kinase (WAK)-like protein, and *ORF13*, encoding a receptor-like kinase protein, were significantly upregulated in CX315 following *Xoo* inoculation. While *ORF9* is predicted to encode the *Xa4* resistance gene, CRISPR/Cas9-based knockout of *Xa4* did not abolish *Xa50*(*t*)-mediated resistance in the CX315 line, indicating that *Xa50*(*t*) confers resistance in a complementary manner. Transcriptome analysis further revealed that oxidative stress response and immune signaling pathways were enriched in CX315 at 48 hours post-inoculation. Together, these findings highlight the potential of *Xa50*(*t*) as a valuable genetic resource for improving BB resistance in rice, and the transcriptome data provides molecular insight into the BB resistance response.

## 1 Introduction

Bacterial blight (BB), caused by *Xanthomonas oryzae* pv. *oryzae* (*Xoo*), is one of the most devastating diseases in rice production, leading to yield losses exceeding 50% in susceptible varieties (Timilsina et al., 2020; Wang et al., 2025). Genetic resistance offers a sustainable strategy for controlling *Xoo* infection, but pathogen evolution can overcome existing resistance mechanisms (Ji et al., 2016; Xu et al., 2022). Therefore, identifying new resources of resistance and development of genetically resistant cultivars is critical for controlling the disease. A thorough understanding of the genetic basis of resistance is essential to maximize its potential in rice breeding programs (Ji et al., 2018; Jiang et al., 2020). Thus, continuous efforts to discover new resistance genes, particularly those effective against emerging *Xoo* pathotypes, remained a priority.

Germplasm collections, including wild relatives, traditional landraces and elite accessions, offer a rich source of genetic diversity for resistance breeding efforts (Tirnaz et al., 2022). These materials often harbor unique resistance traits, making them valuable genetic resources for managing emerging pathogen strains and improving the durability of resistance in cultivated varieties. Recent studies have underscored the potential of rice germplasm in uncovering novel trait related and BB resistance genes (Wang et al., 2023). For example, a diverse panel of rice germplasm from China was evaluated and found to contain accessions with broad-spectrum resistance to *Xoo* strains, even in the absence of previously characterized resistance genes (Lu et al., 2021). Moreover, underutilized germplasm has revealed novel quantitative trait loci (QTLs) associated with disease resistance (Fang et al., 2023). These findings emphasize the unexplored potential of germplasm collections in overcoming the limitations of existing resistance genes.

Rice BB resistance is primarily controlled by resistance (*R*) genes that detect pathogens and activate defense responses (Kumar et al., 2020). Over the past decades, intensive efforts in rice genetics have led to the identification and functional characterization of multiple *R* genes through approaches such as positional cloning, genome-wide association studies (GWAS), mutant screening, and transcriptome profiling. These *R* genes operate via a range of mechanisms, including direct or indirect recognition of pathogen effectors, activation of transcriptional reprogramming, and modulation of defense-related hormone pathways. For example, *Xa21* encodes a receptor-like kinase that perceives conserved pathogen-associated molecular patterns, while *Xa23* and *Xa27* act as executor genes triggered by pathogen effectors to induce localized cell death. To enhance resistance durability and counteract pathogen evolution, gene pyramiding strategies have been employed to combine multiple *R* genes such as *Xa4*, *Xa21*, and *Xa23* into elite cultivars (Yugander et al., 2018; Wang et al., 2020). This has led to the development of rice varieties with broad-spectrum and long-lasting resistance. Another class, wall-associated kinase (WAK)-type *R* genes have shown strong potential for inclusion in such pyramiding strategies due to their unique extracellular sensing and broad defensive functions (Dossa et al., 2020). WAK family members are promising targets for understanding BB resistance and improving rice cultivars.

WAKs have emerged as important *R* genes in plant immunity, acting as molecular guards that monitor cell wall integrity during pathogen attack (Delteil et al., 2016; Harkenrider et al., 2016). These proteins integrate extracellular signals of pathogen invasion with intracellular defense responses, making them essential for both disease resistance and stress adaptation (Stephens et al., 2022). The *Xa4* gene, one of the most widely deployed BB resistance genes in rice (Luo et al., 2012), encodes a WAK-like protein that provides durable resistance to multiple *Xoo* strains and enhances resistance by reinforcing the cell wall remodeling cellulose synthesis (Hu et al., 2017). However, while significant progress has been made in understanding its role in defense, the molecular mechanisms and functional diversity within the WAK family remain underexplored, highlighting the need for further investigation into their potential for improving both biotic and abiotic stress tolerance.

Recent advances in genome editing, particularly CRISPR/Cas9 technology, have revolutionized functional genomics by enabling precise knockout of candidate genes to validate their role in disease resistance (Langner et al., 2018). Coupled with high-throughput RNA sequencing (RNA-seq), these tools allow comprehensive profiling of transcriptional changes in response to pathogen infection and gene disruption (Du et al., 2024). This integrative approach provides deep insights into the molecular mechanisms underlying resistance, helping to distinguish gene-specific effects from broader immune responses. In present study, we utilized CRISPR-mediated knockout of the *Xa4* gene and transcriptome analyses to dissect the independent function of the novel resistance gene *Xa50(t)* in the CX315 germplasm.

Our research screened rice germplasm and identified rice germplasm accession CAAS-11X315 (CX315), which exhibited strong resistance to multiple *Xoo* isolates. To fine map the genetic resistance, we utilized F_2_ population from CX315 × IR24 and fine-mapped the resistance gene to a 147.7 kb region on chromosome 11 and tentatively names it as X*a50(t)*. Quantitative real time PCR (qPCR) and RNA-seq validation showed three genes significantly upregulated after *Xoo* inoculation. Transcriptome analysis revealed enrichment of oxidative stress and immune signaling pathways at 48 hours post-inoculation. Importantly, knockout of *Xa4* did not affect *Xa50(t)*-mediated resistance, indicating *Xa50(t)* acts independently of *Xa4*.

## 2 Material and methods

### 2.1 Plant materials and growth conditions

The rice germplasm line CAAS-11308X315 (CX315) was sourced from the Germplasm Resources Center of CAAS and identified through systematic resistance screening against diverse Xoo strains under controlled inoculation conditions. Two susceptible rice cultivars, IR24 and JG30 used from laboratory-maintained materials as controls in comparison with the resistant line CX315. Statistical analysis of the data was performed using a two-tailed Student’s t-test. Plants were cultivated at CAAS experimental fields located in Beijing and Sanya, Hainan, under net house or open field conditions, with conventional water and fertilizer management practices.

### 2.2 Bacterial strains and inoculation

The *Xoo* strains used to assess the resistance of CX315 to BB included races from China and other countries. These strains included PXO61, PXO86, PXO79, PXO71, PXO112, PXO99_A_, PXO280, PXO339, and PXO341 from the Philippines; T7174, T7147, and T7133 from Japan; KXO85 and KXO576 from Korea; and HLJ72, HB17, NX42, ZHE173, GD1358, LN57, JS49-6, GX15-2, Yun17-3, LN44, and others from China. *Xoo* strains were cultured in NA medium (Polypeptone 5g/L, sucrose 10g/L, yeast extract 1g/L and beef extract 3g/L, Bacto agar 15g/L (for solid media)) at 28°C for three days, then resuspended in sterile distilled water and maintained OD_600_ of 1.0 (measured using Eppendorf spectrometer 1000). Inoculation was performed using either the leaf-clipping method or needleless syringe infiltration, with lesion lengths measured at 14 days post-inoculation (dpi) for leaf clipping and 5 dpi for infiltration.

### 2.3 Genetic analysis and fine mapping of *Xa50*(t)

The susceptible rice cultivar IR24 was crossed with the resistant germplasm line CX315 to develop an F_2_ mapping population through selfing of heterozygous F_1_ plants. Both F_1_ and F_2_ populations were phenotypically screened for resistance or susceptibility following inoculation with T7174 strain. Segregation patterns in the F_2_ population were analyzed using the Chi-square test (χ²)to determine the genetic basis of resistance. Polymorphic insertion-deletion (InDel) markers were developed based on the Shuhui498 reference indica genome (https://mbkbase.org/R498/) and used to identify genetic differences between the parents IR24 and CX315 (**Supplementary Table 2**). Specific primers targeting these polymorphic regions were designed using the Shuhui498 genome sequence. Genomic DNA was extracted from F_2_ individuals using the CTAB method. PCR amplification with polymorphic InDel markers was performed on these DNA samples, and PCR products were resolved by electrophoresis on 5% agarose gels to detect marker segregation. Linkage analysis was then conducted to map candidate genes associated with resistance.

### 2.4 RNA extraction and quantitative real-time PCR

Total RNA was extracted from plant samples using the Trizol reagent according to the manufacturer’s protocol (Rio et al., 2010). The extracted RNA was then reverse-transcribed into first-strand cDNA using the TIANScript-II RT Kit (Tiangen Biotech, Beijing). Quantitative real-time PCR (qRT-PCR) was performed using the Taq Pro Universal SYBR qPCR Master Mix (Vazyme) on an ABI 7500 Real-Time PCR System (Thermo Fisher Scientific, USA). The sequences of primers used for qRT-PCR are given in **Supplementary Table 5**. The 20μL reaction mixture contained 10μL of 2X SYBR Master Mix, 0.4μL each of forward and reverse primer, 2μL of cDNA template, and 7.2μL of nuclease-free water. The PCR program consisted of an initial denaturation at 95°C for 3 minutes, followed by 40 cycles of 95°C for 5 seconds and 60°C for 34 seconds. Relative gene expression levels were calculated using the 2^-ΔΔCt^ method (Livak and Schmittgen, 2001), with the rice Actin gene serving as the internal reference.

### 2.5 Development of *Xa4*-KO CRISPR vector and transformation

Target sequences within the exon of the *Xa4* gene were selected using the TargetDesign tool (http://skl.scau.edu.cn/targetdesign/). These sequences were ligated into sgRNA expression cassettes via overlapping PCR to generate two sgRNAs, sgRNA-U6a-T1 and sgRNA-U6b-T2 (**Supplementary Table 4**). The sgRNA cassettes were cloned into the binary vector pYLCRISPR-Cas9-*Xa4*-KO using Golden Gate cloning (Ma et al., 2015). Agrobacterium-mediated transformation was performed to introduce the CRISPR construct into rice calli. Genomic DNA was extracted from transformed plant leaves using the CTAB method and used as template for PCR amplification with *Xa4*-KO-seq primers listed in **Supplementary Table 4**. Sanger sequencing of the PCR products used for identification of knockout mutations in *Xa4*.

### 2.6 RNA-sequencing

Total RNA was extracted from rice leaf tissues using the Trizol reagent, and RNA quality was assessed using a Nanodrop spectrophotometer and Bioanalyzer. RNA libraries were prepared using the TrueSeq RNA Library Prep Kit (Illumina) with fragmentation, reverse transcription, and amplification steps, followed by sequencing on an Illumina NovaSeq 6000 platform with paired-end 150 bp reads. Raw reads were processed to remove low-quality bases and adapter sequences using Trimmomatic and aligned to the rice reference genome *Shuhui498* using STAR. Gene expression levels were quantified with feature Counts and analyzed for differential expression using DESeq2, considering a fold change ≥ 2 and an FDR < 0.05. Functional annotation of differentially expressed genes was performed using the NCBI Nr and GO databases, with KEGG pathway analysis to identify enriched biological processes. RNA-seq data was submitted to NCBI SRA database under project PRJNA1209632.

## 3 Results

### 3.1 CX315 confers broad-spectrum and genetically controlled resistance to *Xoo*

**Figure 1:**
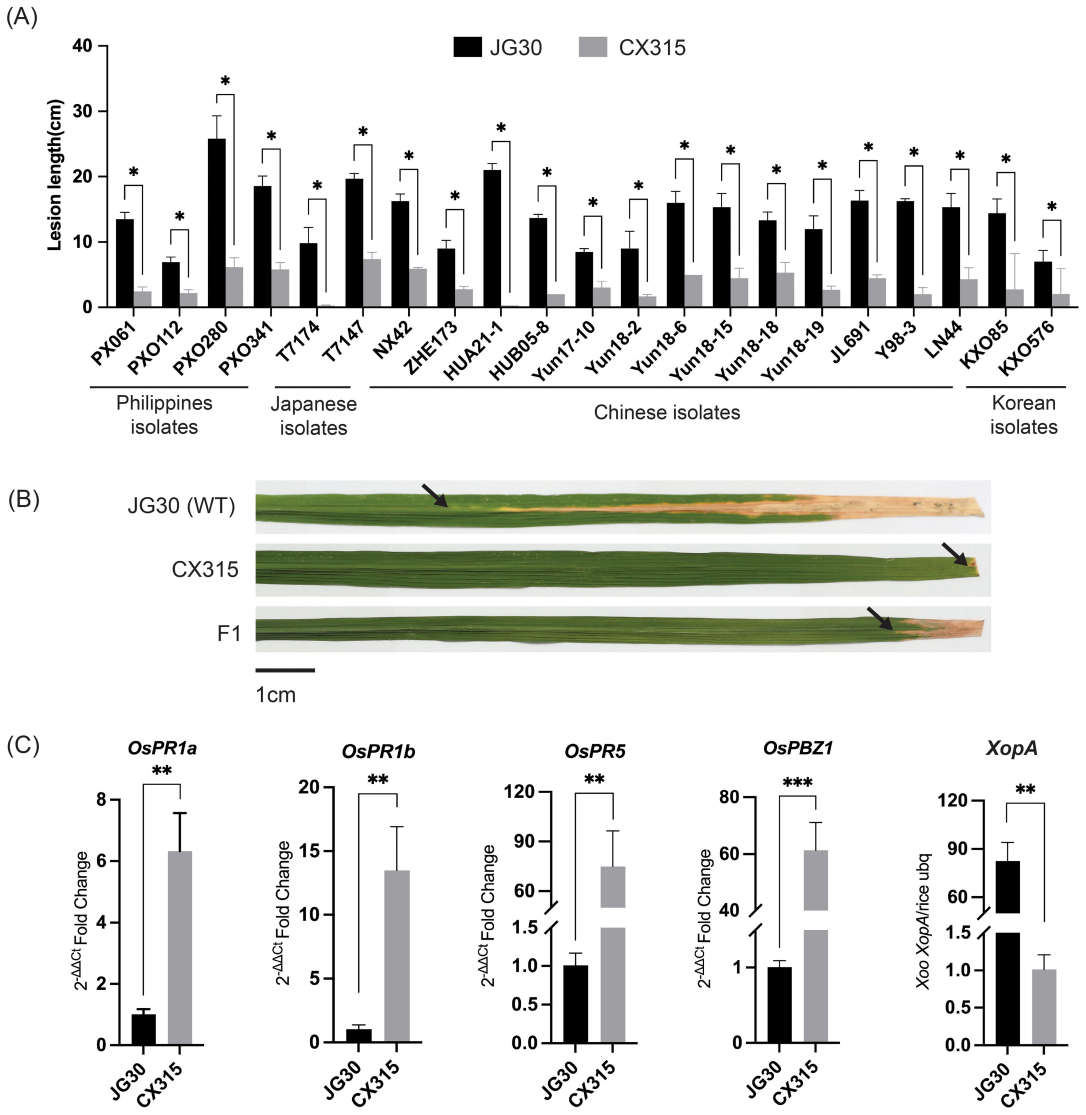
Phenotypic and molecular characterization of resistance conferred by CX315 to *Xoo*. **(A)** Lesion length comparison between the resistant line CX315 and the susceptible parent JG30 across different *Xoo* strains. CX315 exhibited significantly reduced lesion lengths compared to JG30 for all tested strains. Asterisks (*) indicate significant differences (*p< 0.05, **p< 0.01) between CX315 and JG30 as determined by Student’s *t*-test. **(B)** Representative images of lesion development on leaves of JG30, CX315, and the F1 hybrid plants 14 days post inoculation with *Xoo* strain T7174. JG30 leaves exhibit severe lesions, while CX315 and F1 plants demonstrate resistance with shorter lesions. Black arrows highlight the lesion sites, and a scale bar of 1 cm is provided for reference. **(C)** Differential expression of defense-related genes (*OsPR1a, OsPR1b, OsPR5, OsPBZ1*) and the *Xoo* effector gene *XopA* in JG30 and CX315 after inoculation with T7174. CX315 shows significantly higher upregulation of these genes compared to JG30, indicating activation of defense pathways. Conversely, the reduced abundance of XopA in CX315 suggests a significantly lower bacterial load in the resistant line.

Initial screening of germplasm lines identified CX315 as highly resistant line to *Xoo* strains from the Philippines, China, Japan, and Korea. To ascertain the genetic resistance, CX315 and the susceptible cultivar JG30 were inoculated at the tillering stage with 21 representative *Xoo* strains. CX315 exhibited significantly robust resistance to all strains, with lesion lengths ranging from 0.1 to 6 cm, whereas JG30 showed consistent susceptibility, with lesion lengths exceeding 7 cm (**Figure 1A**). These findings highlight CX315 as a promising source of broad-spectrum resistance for bacterial blight in rice.

To determine whether CX315’s resistance is genetically controlled, we crossed CX315 (♂) and the susceptible cultivar IR24 (♀), and the resulting F_1_ plants were evaluated after inoculation with the *Xoo* strain T7174. The F_1_ plants exhibited resistance comparable to CX315, as evidenced by shorter lesion lengths (**Figure 1B**). Evaluation of F_2_ population informed 1,767 plants exhibited resistance, while 555 plants were susceptible, fitting a 3:1 Mendelian segregation ratio (χ² = 1.4935, *p* = 0.222), thereby confirming that resistance in CX315 is controlled by a single dominant gene (**Supplementary Table 1**). To further validate that this resistance is not only genetically inherited but also associated with active defense responses, we analyzed the expression of key pathogenesis-related (PR) genes following pathogen challenge. PR genes are molecular markers of plant immune activation, and their upregulation reflects the activation of basal and systemic defense pathways. qRT-PCR results showed significant induction of *OsPR1a*, *OsPR1b*, *OsPR5*, and *OsPBZ1* in CX315 compared to JG30 (**Figure 1C**), indicating that CX315 confers a strong defense response upon infection. Additionally, the bacterial population of *Xoo* in CX315 was significantly lower than in JG30, as shown by reduced expression of the bacterial effector gene *XopA* (**Figure 1C**). These findings collectively support the hypothesis that CX315 harbors a genetically controlled resistance mechanism that actively suppresses pathogen proliferation through enhanced immune signaling.

### 3.2 Fine mapping of *Xa50(t)* identifies a candidate region on chromosome 11

**Figure 2:**
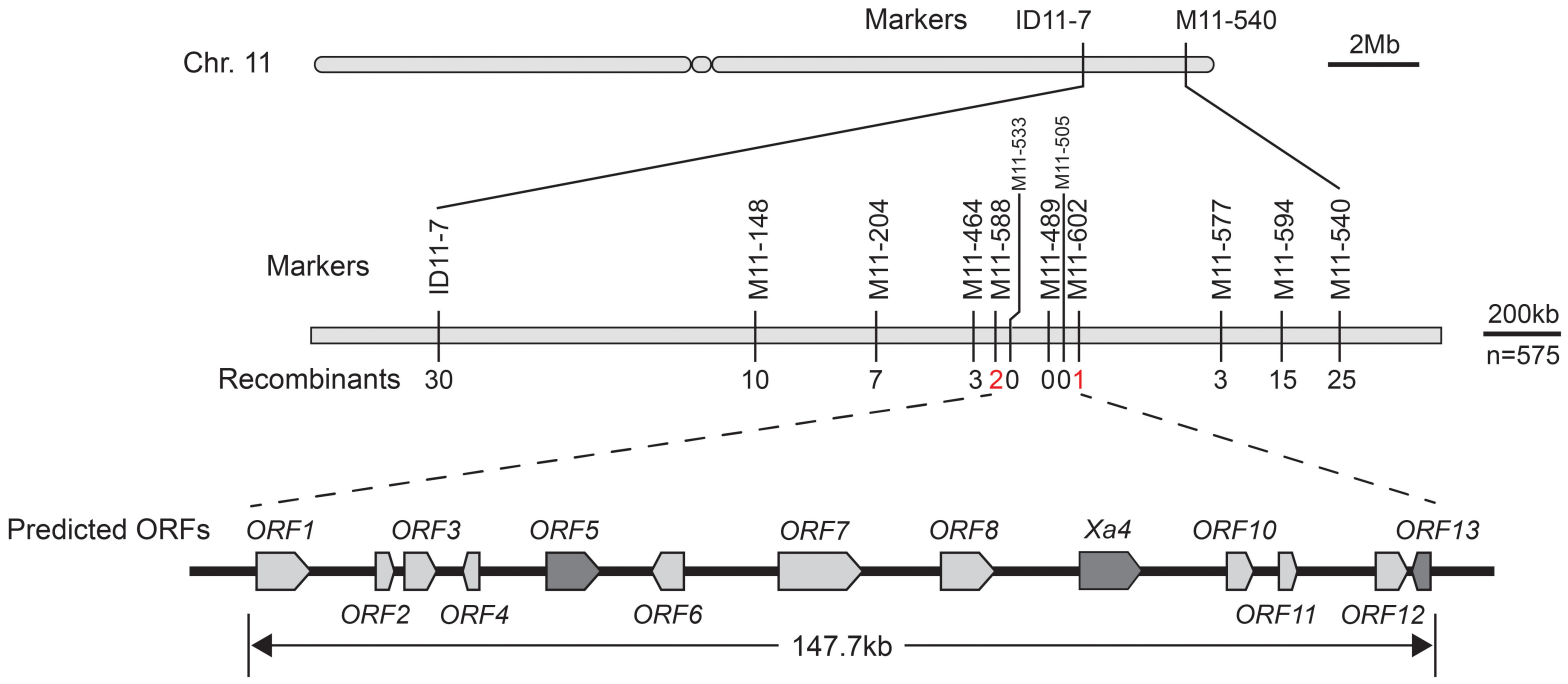
Fine mapping of *Xa50(t)* on Chromosome 11. Genetic mapping of the resistance gene *Xa50*(t) using recombinants from the F2 population. The region was narrowed down to a 147.7 kb interval flanked by markers M11–588 and M11–602 on Chromosome 11. The recombination frequency is displayed for key markers, and the final co-segregating marker (M11-489) is indicated in red. Within the interval, 14 predicted open reading frames were identified.

To fine-map the *R* gene in CX315 and elucidate the genetic basis of its resistance, an F_2_ mapping population was generated by selfing heterozygous F_1_ plants derived from a cross between CX315 and IR24. This population was phenotyped for resistance to the *Xoo* strain T7174. To locate the resistance locus, 256 insertion-deletion (InDel) markers distributed across all 12 rice chromosomes were screened for polymorphism between CX315, IR24, and their F_2_ progeny. Totally, 112 markers were polymorphic and used for genotyping. Linkage analysis based on recombination frequencies revealed that markers ID11–7 and M11-540, located on chromosome 11, were tightly associated with the resistance phenotype, with recombination events observed in 30 and 25 individuals, respectively (**Figure 2**). Subsequent fine mapping narrowed the candidate interval to a 147.7 kb region between markers M11–588 and M11-602, with M11–489 showing complete co-segregation with the resistance trait. This high-resolution mapping region harbors 13 predicted open reading frames (ORFs) according to the reference genome (Shuhui498). Gene annotation within the 147.7 kb mapping interval on chromosome 11 identified a high-confidence candidate region for the novel resistance gene *Xa50(t)* (**Supplementary Table 3**). While *ORF9* is predicted to be *Xa4* gene locus we named *ORF9* to *Xa4* subsequently. The presence of WAK, receptor-like kinase, and leucine-rich repeat encoding ORFs within the candidate region warrants further investigation to precisely identify the *Xa50(t)* gene.

### 3.3 Transcriptome analysis of CX315 and JG30 in response to *Xoo*

**Figure 3:**
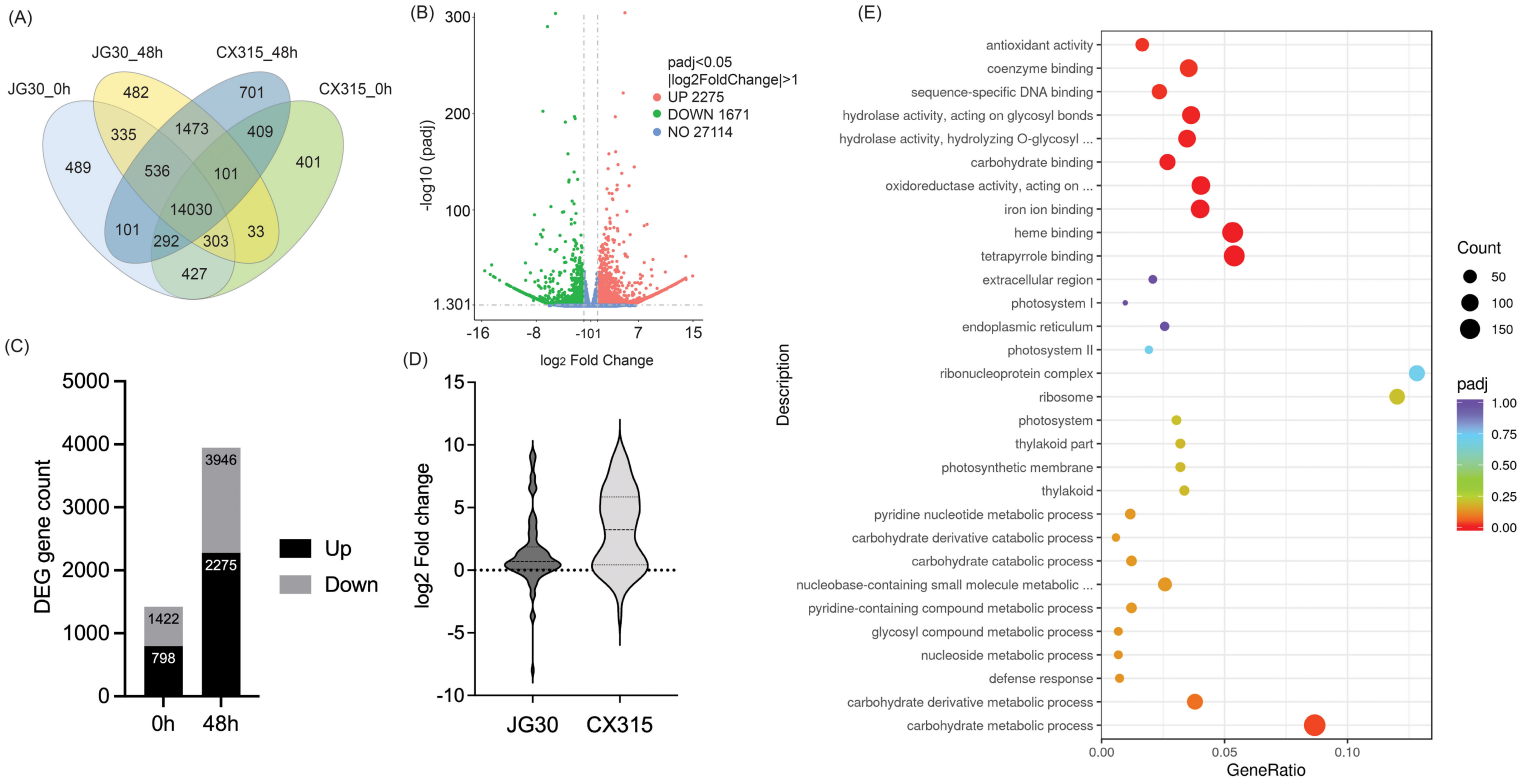
Differential gene expression and enrichment analysis in CX315 and JG30 during *Xoo* infection. **(A)** Venn diagram representing overlapping and unique differentially expressed genes (DEGs) across CX315 and JG30 at 0 hours (0h) and 48 hours post-inoculation (48h). CX315 shows a unique set of DEGs at 48h, highlighting its distinct transcriptional response to Xoo infection. **(B)** Bar graph showing the distribution of upregulated (black) and downregulated (gray) DEGs in CX315 compared to JG30 at baseline (0h) and post-inoculation (48h). The number of DEGs increases significantly at 48h, reflecting an enhanced transcriptional reprogramming in CX315 during infection. **(C)** Volcano plot of DEGs between CX315 and JG30 at 48h. Genes with significant upregulation (log_2_FoldChange > 1, *padj* < 0.05) are shown in pink, while significantly downregulated genes are shown in green. Non-significant genes are represented in blue. This plot highlights key genes strongly involved in defense and metabolic processes. **(D)** Violin plot illustrating the distribution of log_2_ fold change in WAK gene expression at 48h between CX315 and JG30. **(E)** Gene Ontology (GO) enrichment analysis of DEGs in CX315 at 48h. Significantly enriched GO terms are shown for biological processes, molecular functions, and cellular components. The size of the dots indicates the number of DEGs associated with each term, while the color represents statistical significance (adjusted p-value).

Transcriptome profiling revealed significant differential gene expression (DEG) patterns in CX315 and JG30 at both 0 and 48 hpi with *Xoo* strain T7174, highlighting key molecular mechanisms of resistance. Functional enrichment analyses emphasized the activation of defense-related pathways in CX315, along with suppression of susceptibility-associated processes. A total of 6,221 differentially expressed genes (DEGs) were identified between CX315 and JG30 at 48 hpi, with 2,275 upregulated and 3,946 downregulated in CX315 (**Figure 3C**). The Venn diagram (**Figure 3A**) shows the distribution and overlap of DEGs across genotypes and timepoints, revealing a distinct set of CX315-specific DEGs at 48 hpi. The volcano plot (**Figure 3B**) illustrates the magnitude of differential expression, highlighting genes with strong pathogen-responsive activation or repression.

At baseline (0 hpi), 2,220 DEGs were identified between CX315 and JG30, including 798 upregulated and 1,422 downregulated genes in CX315. GO enrichment of these genes indicated that upregulated transcripts were predominantly involved in oxidative stress response and carbohydrate derivative metabolic processes, suggesting that CX315 maintains a primed defense state even prior to infection. Pathway analysis further revealed enrichment in phenylpropanoid biosynthesis and MAPK signaling—two key components of plant immune responses.

At 48 hpi, GO enrichment of CX315 DEGs demonstrated activation of defense-related processes, including “response to biotic stimulus,” “oxidoreductase activity,” and “cell wall organization,” while downregulated genes were enriched in photosynthesis-related pathways (**Figure 3E**). This shift reflects a strategic reallocation of metabolic resources from growth to defense under pathogen attack. In line with this, members of the wall-associated kinase (WAK) family showed higher expression in CX315 compared to JG30 at 48 hpi (**Figure 3D**), consistent with their involvement in *Xa50(t)*-mediated immune signaling.

Temporal comparison within CX315 (0 h *vs*. 48 hpi) identified 5,321 DEGs, of which 2,491 were upregulated and 2,830 were downregulated post-inoculation. Enriched biological processes among upregulated genes included oxidative stress and chitin metabolic responses, while downregulated transcripts were associated with photosynthetic pathways such as photosystem I and thylakoid membrane components.

Overall, these findings demonstrate that CX315 mounts a rapid and coordinated transcriptional defense in response to *Xoo*, involving the activation of key immune pathways and suppression of susceptibility-associated and energy-intensive metabolic processes. This dynamic transcriptional reprogramming provides insight into the molecular mechanisms by which *Xa50(t)* confers enhanced resistance to bacterial blight.

### 3.4 Differential expression of candidate genes in response to *Xoo* inoculation

**Figure 4:**
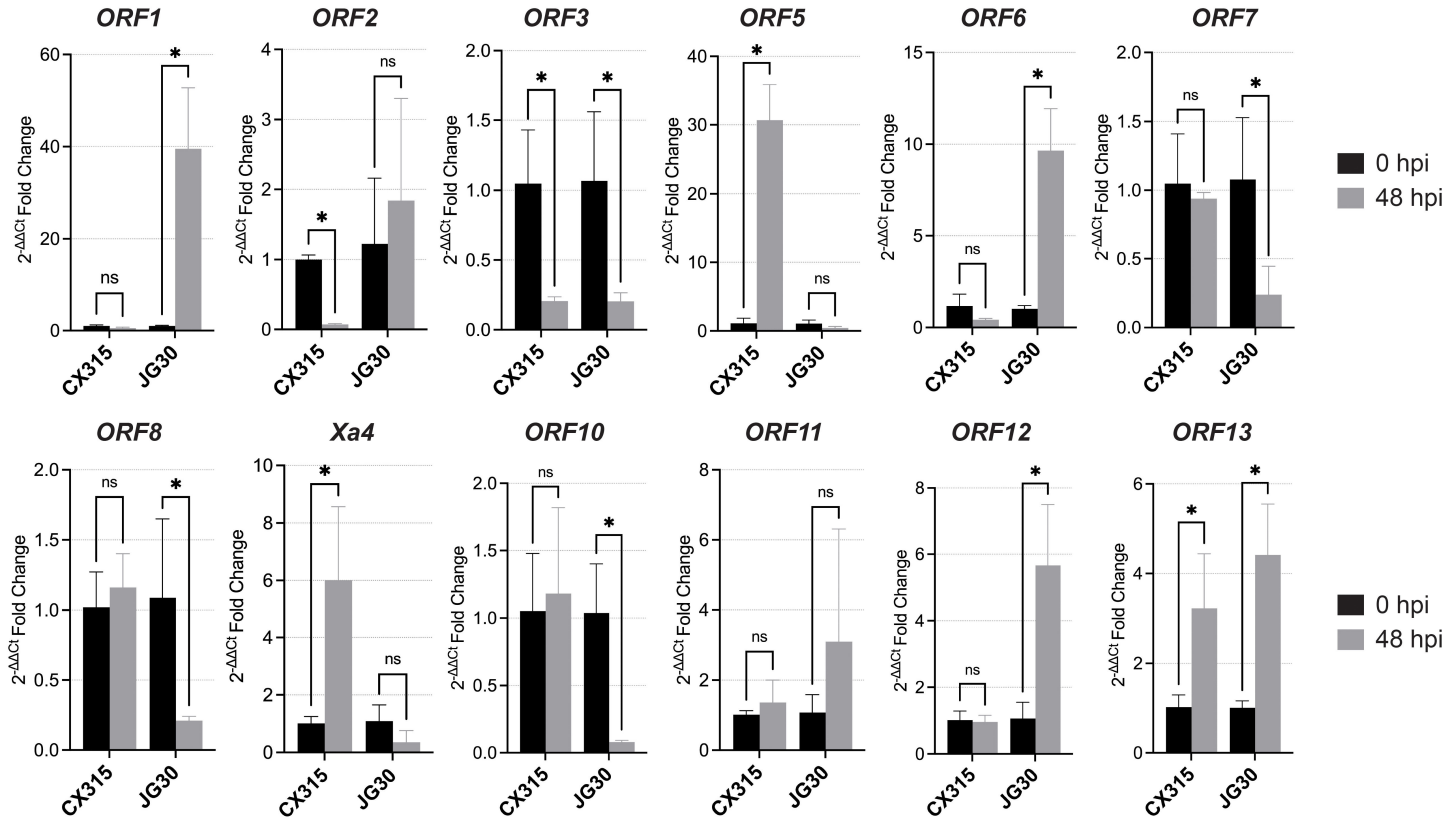
Expression analysis of the 12 candidate ORFs in CX315 (resistant) and JG30 (susceptible) plants at 0 and 48 hours post inoculation (hpi) with *Xoo* strain T7174. Significant upregulation was observed in several ORFs (e.g., *ORF5*, *Xa4* and *ORF13*) in CX315, while JG30 exhibited no significant induction. Statistical significance was determined using a Student’s t-test (*p<0.05; ns, not significant).

It was indicated that pathogen-triggered induction of *Xa4* expression contributes to its disease resistance (Hu et al., 2017). To prioritize candidate genes within the 147.7 kb interval linked to *Xa50(t)*, qRT-PCR was conducted on all 13 predicted ORFs in CX315 and the susceptible cultivar JG30 at 0- and 48-hours post-inoculation with *Xoo* strain T7174. Three genes *ORF5, Xa4*, and *ORF13* were significantly upregulated in CX315 at 48 hpi, while expression of *ORF5* and *Xa4* remained unchanged in JG30 (**Figure 4**). Interestingly, *ORF13* displayed moderate induction in JG30, suggesting it may be partially responsive to pathogen inoculation even in the absence of functional resistance (**Figure 4**). This pathogen-responsive expression in the resistant background suggests their potential roles in *Xa50(t)*-mediated defense. The expression of *ORF4* could not be reliably determined, due to technical limitations in primer design.

*ORF5* encode wall-associated kinase (WAK)-like proteins, which act as sensors of cell wall perturbation or pathogen-derived signals, initiating immune responses. *ORF13* encodes a receptor-like kinase (RLK) with WAK-related domains, suggesting a role in downstream signal transduction or receptor complex formation. The co-induction of these kinase-domain genes points to a multilayered signaling cascade contributing to CX315 resistance. Their selective activation in response to *Xoo* highlights them as promising candidates underlying *Xa50(t)* and warrants further functional analysis.

### 3.5 *Xa50(t)* provide distinct resistance than *Xa4*

**Figure 5:**
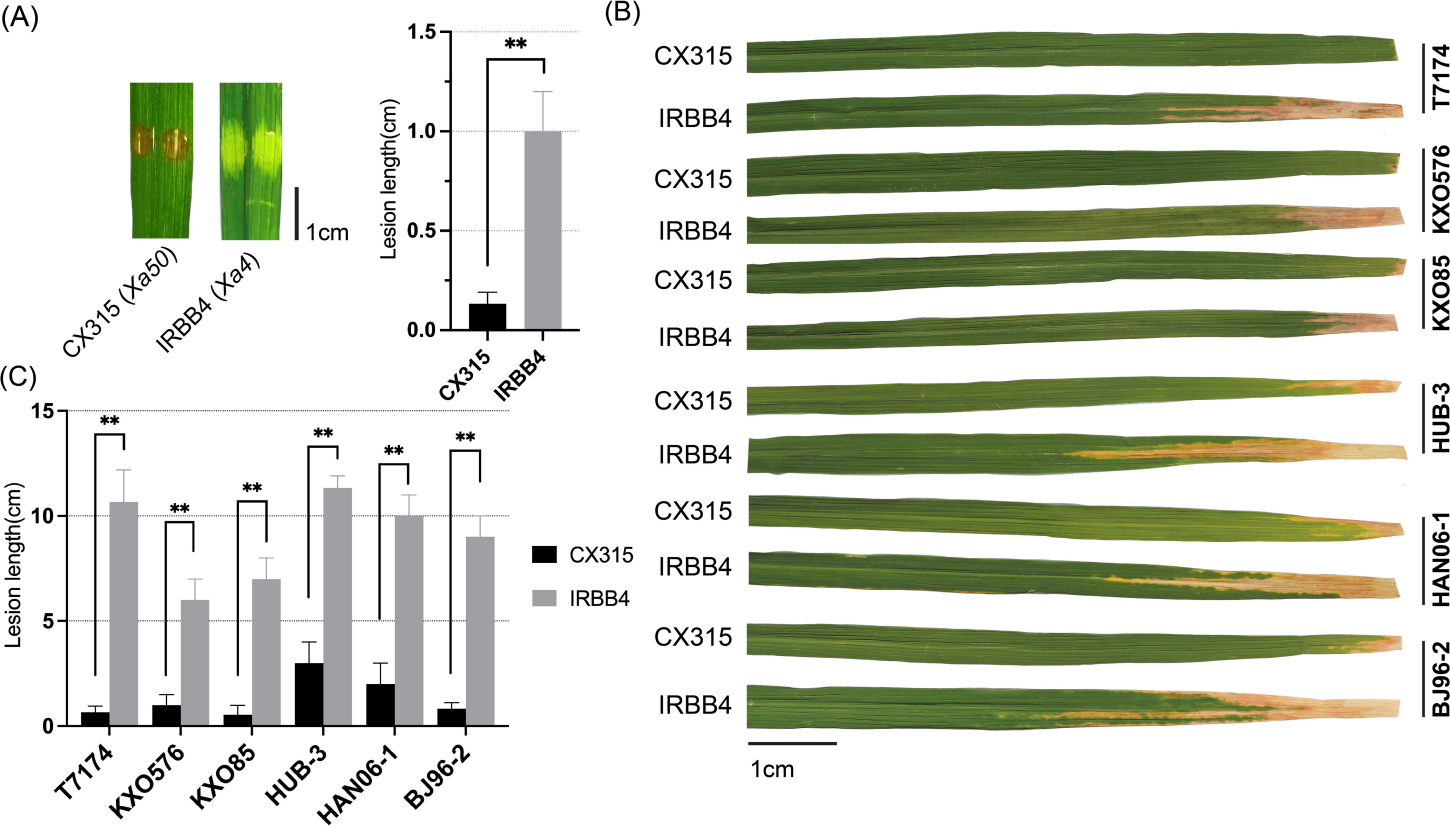
*Xa50(t)* confers resistance independently of *Xa4* in rice. **(A)** Comparative resistance response of CX315 (*Xa50(t)*) and IRBB4 (*Xa4*) following seedling-stage inoculation with *Xoo* strain T7174. CX315 displayed significantly shorter lesion lengths than IRBB4. Representative leaf images and lesion quantification are shown. Data represent mean ± SD (n = 3); p < 0.01 (Student’s t-test). **(B)** Resistance profiles of CX315 and IRBB4 against six diverse *Xoo* strains at the tillering stage. Significant differences in lesion lengths between the two lines were observed for several strains, indicating differential resistance spectra. **(C)** Phenotype of leaves shown at right, with lesion length bar = 1cm. Data represent mean ± SD (n = 3). The asterisks (**) indicate a statistically significant difference (p < 0.01, Student’s *t*-test).

Since the fine-mapped interval of *Xa50(t)* overlapped with the known *Xa4* locus, comparative pathotyping was performed to determine whether *Xa50(t)* is allelic to *Xa4* or confers resistance through an independent mechanism. For this purpose, the *Xa4*-containing line IRBB4 and the *Xa50(t)*-harboring line CX315 were inoculated with the *Xoo* strain T7174 at the seedling stage and further challenged with a panel of diverse *Xoo* strains at the tillering stage. At the seedling stage CX315 exhibited a hypersensitive-like response and significantly shorter lesions compared to IRBB4 (**Figure 5A**). At the tillering stage, the resistance responses significantly varied between CX315 and IRBB4 for several strains, indicating that the underlying mechanisms differ between *Xa50(t)* and *Xa4* (**Figures 5B, C**).

**Figure 6:**
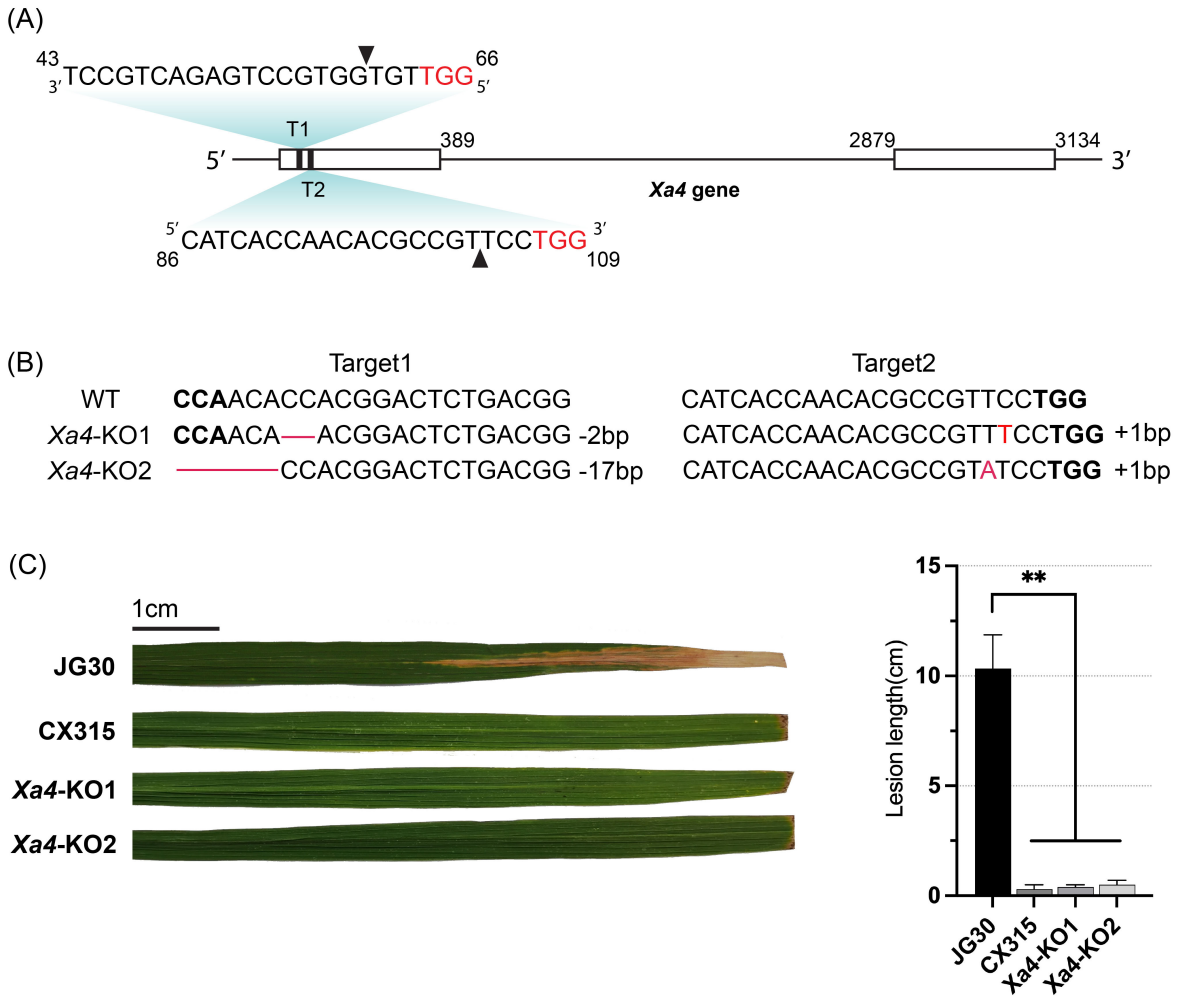
Knockout of *Xa4* do not compromise resistance of CX315. **(A)** Schematic representation of the *Xa4* gene and CRISPR/Cas9 target sites. Two target sequences (T1 and T2) located in the first exon were used for editing. Protospacer adjacent motif (PAM) sites are highlighted in red; cleavage sites are indicated by arrowheads. **(B)** Sanger sequencing of *Xa4-*KO1 and *Xa4*-KO2 lines confirmed small insertions/deletions (indels) at target loci. WT: wild-type JG30 sequence; red dashes denote deleted bases; PAM sequences in bold. **(C)** Lesion length analysis of JG30, CX315, and *Xa4*-KO lines at 14 days post-inoculation with *Xoo* strain T7174. The *Xa4*-KO lines maintained strong resistance, similar to CX315, whereas JG30 remained susceptible. Data are shown as mean ± SD (n = 3); p < 0.01 (Student’s *t*-test). Representative infected leaves are shown; scale bar = 1 cm.

To confirm the presence of *Xa4* in the CX315 background, we amplified and sequenced its coding region, which verified that *Xa4* is intact in CX315 and suitable for targeted editing. To further validate whether *Xa50*(*t*) acts independently of *Xa4*, we employed CRISPR/Cas9-mediated knockout of *Xa4* in the CX315 background. Two guide RNAs targeting the first exon of *Xa4* were designed (**Figure 6A**), and the sgRNA expression cassettes were cloned into a CRISPR/Cas9 binary vector. Two homozygous T_1_ transgenic lines were obtained by Sanger sequencing confirmed indel mutations at the targeted sites, resulting in frameshift knockouts of *Xa4* (**Figure 6B**, **Supplementary Figure S1**). Protein alignment confirmed that both *Xa4*-KO lines produce truncated proteins due to premature stop codons (**Supplementary Figure S2**). T_1_ homozygous knockout plants were inoculated with *Xoo* strain T7174 at the maturity stage. These *Xa4*-KO lines retained resistance phenotypes similar to the wild-type CX315 (**Figure 6C**), demonstrating that *Xa50(t)*-mediated resistance remains functional in the absence of *Xa4*.

Together, the phenotypic divergence between IRBB4 and CX315, along with the retained resistance observed in *Xa4*-knockout lines, confirms that *Xa50(t)* operates independently and represents a functionally distinct resistance gene. These findings underscore the potential of *Xa50(t)* as a valuable genetic resource for breeding broad-spectrum and durable resistance in rice. However, the current mapping interval contains multiple candidate resistance genes, which prevents definitive identification of *Xa50(t)* at this stage. Further studies aimed at dissecting the regulatory networks and functional mechanisms associated with *Xa50(t)* will be essential to elucidate how this novel *R* gene contributes to immune specificity and complements existing resistance pathways in rice.

## 4 Discussion

The identification and mapping of *Xa50(t)* in this study provide a strong foundation for exploring BB resistant resource material in rice and represent a critical step towards breeding BB-resistant varieties. Map-based cloning facilitated the fine-mapping of *Xa50(t)* to a 147.7 kb region on chromosome 11, overlapping with the known resistance gene *Xa4*. Interestingly, this overlap revealed that the resistance observed in the germplasm CX315 was mediated by *Xa50*(t), while *Xa4* was also present but functionally distinct. While, *Xa4* gene belong to WAK-like family, which plays a central role in plant immunity by sensing cell wall perturbations and activating defense responses. Despite *Xa4* and *Xa50(t)* proximity and functional similarities, the results of this study highlight the unique and complementary roles of *Xa50(t)* and *Xa4* in combating *Xoo*.

The resistance responses of *Xa50(t)*-containing CX315 and *Xa4*-containing IRBB4 to a panel of several *Xoo* strains underscore the distinct and non-redundant nature of these genes. For example, both genes conferred resistance to strains like T7174, KXO576, and KXO85, but with significant differences in lesion lengths, with *Xa50*(t)-mediated resistance showing much shorter lesions (*p* < 0.01 and *p* < 0.05). This suggests that *Xa50*(t) provides a more robust resistance to certain strains compared to *Xa4*.

Transcriptomic analysis provided additional insights into the distinct defense mechanisms of *Xa50(t)*. Upon *Xoo* inoculation, significant differences were observed in the gene expression profiles of CX315 (*Xa50(t)*) and IRBB4 (*Xa4*). Genes involved in phenylpropanoid, and flavonoid biosynthesis pathways were strongly upregulated in CX315, suggesting that *Xa50(t)* activates specific secondary metabolic pathways to reinforce cell walls and produce antimicrobial compounds. In contrast, *Xa4*-mediated resistance relies more on cell wall pectin sensing and brassinosteroid-regulated cell elongation. The differential expression of transcription factors, kinase-responsive genes, and hormone-responsive genes in CX315 highlights the unique regulatory network activated by *Xa50*(t). Notably, the upregulation of genes associated with ethylene and jasmonic acid signaling pathways indicates that *Xa50*(t) employs a broader hormonal crosstalk compared to *Xa4*, which predominantly regulates brassinosteroid signaling.

The structural and functional diversity within the WAK family, as exemplified by *Xa4*, underscores their central role in plant defense. WAKs serve as molecular bridges between the extracellular cell wall and intracellular signaling pathways, monitoring cell wall integrity and triggering immune responses upon pathogen attack. This divergence likely reflects an evolutionary strategy to expand the recognition repertoire of rice against a rapidly evolving pathogen like *Xoo*. Comparative studies of WAKs in rice and other crops could provide deeper insights into their evolutionary origins and functional diversification.

From a practical perspective, the distinct resistance profiles of *Xa50(t)* and *Xa4* have significant implications for rice breeding. The ability of *Xa50(t)* to provide robust resistance to a wide range of *Xoo* strains makes it a valuable genetic resource for developing BB-resistant rice varieties. Moreover, the complementary resistance patterns of *Xa50(t)* and *Xa4* highlight the potential for gene pyramiding to enhance the durability and breadth of resistance. Marker-assisted selection using molecular markers linked to *Xa50(t)* can accelerate its deployment in breeding programs. Furthermore, combining *Xa50(t)* with other resistance genes, including those targeting different pathogen effectors or stages of infection, could provide synergistic effects, reducing the risk of resistance breakdown due to pathogen adaptation.

Recent studies have highlighted the importance of structural variation (SV) including insertions, deletions, transpositions, and presence/absence variations in shaping disease resistance loci in rice. The *Xa4* locus, in particular, resides within a WAK-rich genomic region known for high SV frequency and haplotype diversity across cultivated and wild rice accessions (Hu et al., 2017; Zhao et al., 2018). Analysis from the 3K Rice Genomes Project revealed extensive structural remodeling at resistance gene clusters, including transposon insertions, promoter rearrangements, and gene copy number variation, all of which can influence gene expression and specificity. The distinct resistance profile of CX315, despite overlapping the *Xa4* locus, suggests that it harbors a unique haplotype shaped by such SV events. This structural differentiation likely underlies the independent function of *Xa50(t)*, allowing it to confer resistance distinct from *Xa4*. Our findings reinforce the concept that SV is a key driver of novel *R* gene emergence and diversification, and that SV-rich regions such as the *Xa4* locus are hotspots for mining durable resistance alleles in rice breeding programs. Due to the complexity of this region and the lack of long-read genome data for CX315, we were unable to reconstruct the complete sequence. Therefore, candidate gene identification relied on transcriptome profiling and qRT-PCR expression analysis. Future work involving high-resolution sequencing of this locus will be necessary to resolve the precise gene structure and distinguish *Xa50(t)* from closely linked homologs such as *Xa4*.

In conclusion, this study not only advances our understanding of the genetic and molecular basis of BB resistance in rice but also provides practical tools for crop improvement. The distinct roles of *Xa50(t)* and *Xa4* in resistance, their complementary profiles, and their structural differences within the importance of exploring diverse genetic resources for sustainable disease management. The functional validation of *ORF5* and *ORF13* is warranted, and future studies involving gene knockout and overexpression will be essential to resolve their specific roles in *Xa50(t)*-mediated resistance.

Future research should focus on elucidating the molecular interactions between *Xa50(t)* and *Xoo* effectors, as well as exploring its potential role in regulating abiotic stress tolerance, given the multifunctional nature of WAKs. Such efforts will contribute to the broader goal of developing resilient rice varieties capable of withstanding both biotic and abiotic challenges.

## Data Availability

The RNA-seq data have been deposited in the NCBI Sequence Read Archive (SRA) under Project ID PRJNA1209632, with accession numbers SRR31970743, SRR31970744, SRR31971027, and SRR31971028.
