# Supplementary material for: A new bacterial blight resistance gene Xa50(t) in the Xa4 locus confers resistance against Xanthomonas oryzae pv. oryzae in rice
Source: Front Plant Sci. 2025 Aug 29;16:1657476. doi: 10.3389/fpls.2025.1657476 (PMC12427036; doi:10.3389/fpls.2025.1657476)
Supplement: Supplementary file 1 [file DataSheet1.docx]

**Supplementary data:** **A new bacterial blight resistance gene *Xa50(t)* in the *Xa4* locus confers resistance against Xanthomonas oryzae pv. oryzae in rice**

**Supplementary tables**

Supplementary table 1: Chi-Square Analysis for F_2_ Population

Supplementary table 2: InDel polymorphic markers used for fine mapping of *Xa50(t)* gene

Supplementary table 3: Annotated genes on chromosome 11 at 147.7 kb region linked to *Xa50(t)*

Supplementary table 4: *Xa4*-KO targets and knockout plant sequencing primers

Supplementary table 5: qPCR primers used in present study

**Supplementary figures**

Supplementary figure 1: Sanger sequencing results of *Xa4*-KO plants

Supplementary figure 2: Protein sequence alignment of *Xa4* and *Xa4*-KO plants

**Supplementary Table 1. Chi-Square Analysis for F_2_ Population**

| Cross  (Population) | Observed Resistant | Observed Susceptible | Expected Ratio (R:S) | Expected Resistant | Expected Susceptible | Chi-Square (χ^2^)Value | p-Value |
| --- | --- | --- | --- | --- | --- | --- | --- |
| CX315 × IR24 (F_2_) | 1,767 | 555 | 3:1 | 1,741.50 | 580.5 | 1.4936 | 0.22 |

**Supplementary Table 2. InDel polymorphic markers used for fine mapping of *Xa50*(t) gene**

| InDel | Forward sequence | Reverse sequence |
| --- | --- | --- |
| ID11-7 | GCATTCAGCGTTTTCCACTGT | GAAGGAAAGCATTTGTGGGTGA |
| M11-148 | AGCCTCGAGTTCTACTTTGGC | CCTACCACCAGCTCGCTTTAT |
| M11-439 | TCATGAACTATCACGCGTTTGAA | GGATGTGATCTGAGCAGTTGGA |
| M11-443 | GGGAAGGGCAAGTTTGCTTTAG | CGAGCAGATTATTCGGGGTC |
| M11-204 | GCTCGCGAGGGTTTCTATCC | ACTTGTAAGCGACTGCCCAA |
| M11-464 | AGCATTGATGAACTCGTTGCC | CAGGAGGTAACCTTAGAACTGAA |
| M11-588 | CGTTGCTATTCTAATCTCACCTCT | CTTGCATGCCATGCATCTCT |
| M11-533 | GACGAAGAGACCCAGGAGCTG | ACTTCTTTTCCGGAAGCCAGTC |
| M11-481 | TGGACATTACCATCTTCTCAGAGA | CCGCAACATTTATGATGGCACA |
| M11-506 | TGGATGGGGATCACTTGCAG | GGCTCAGCTTTCCTTGGAGG |
| M11-489 | ATCTGAACCTGAACATTACTG | CATTCTGGTAAAAGTATGCATCT |
| M11-505 | GGATGTAGTACTCAATTCAGGATAC | AAAAGTATGCATCTCCGTTGT |
| M11-602 | TAGACGTGTCGCTGGCAATG | TGGAACACTGCTGTTTGGTTCT |
| M11-528 | GGTTGAATTGAATATATCT | ATATAAGCAGCACATCATCTAC |
| M11-462 | TGAACGATTGTTGATCTTTCTTGT | ACAGTTGTTCTTAATTCCTGTGCA |
| M11-577 | CGCGCACACTCACCTGTTTATA | CGGGTCAACCAGATCTTACGG |
| M11-594 | CACCCTAGGCTCCGTAGACAT | GGGTGTTCATAGTGCTTTGACA |
| M11-540 | AATTAAGCTAGCTTCGTTGGCG | GATATGCATGCTGCATTGCCC |

**Supplementary table 3. Annotated genes in the 147.7 kb mapping region on chromosome 11 linked to *Xa50(t)***

| ORF. No. | Gene ID (Shuhui498) | Start and end position | Gene length | Protein domain predicted |
| --- | --- | --- | --- | --- |
| *ORF1* | OsR498G1120740500 | 30708234..30711180 | 2947 | Wall-associated receptor kinase 3 precursor, putative, expressed |
| *ORF2* | OsR498G1120740700 | 30722286..30722550 | 265 | Wall-associated receptor kinase galacturonan-binding domain |
| *ORF3* | OsR498G1120741100 | 30723442..30724773 | 1332 | Serine/threonine/dual specificity protein kinase |
| *ORF4* | OsR498G1120741400 | 30730166..30731170 | 1005 | Uncharacterized |
| *ORF5* | OsR498G1120741700 | 30735670..30740239 | 4570 | EGF-like calcium-binding domain |
| *ORF6* | OsR498G1120742400 | 30744894..30748486 | 3593 | Uncharacterized |
| *ORF7* | OsR498G1120743300 | 30763015..30770958 | 7944 | Wall-associated receptor kinase galacturonan-binding domain |
| *ORF8* | OsR498G1120743900 | 30794787..30798006 | 3220 | Uncharacterized |
| *ORF9*  (*Xa4*) | OsR498G1120744100, | 30811256.. 30816194 | 2121 | Wall-associated kinase |
|  | OsR498G1120744300 |  |  |  |
| *ORF10* | OsR498G1120744500 | 30827148..30828131 | 984 | Leucine-rich repeat-containing N-terminal |
| *ORF11* | OsR498G1120744700 | 30829131..30829585 | 455 | Protein kinase-like domain |
| *ORF12* | OsR498G1120745300 | 30847997..30849322 | 1326 | Serine-threonine/tyrosine-protein kinase catalytic domain |
| *ORF13* | OsR498G1120745000 | 30848272..30850304 | 2033 | Receptor-like protein kinase 5 precursor, putative |

**Supplementary table 4. Primers used for developing and genotyping CRISPR-Cas9 base knockout plant of *Xa4***

| Primer name | Forward primer | Reverse primer |
| --- | --- | --- |
| *Xa4*-KO-seq | GTGGACATCCCCTACCCATTC | GGCGTAGCTACAGGGACTTT |
| OsU6aT1 | gccgTCCGTCAGAGTCCGTGGTGT | aaacACACCACGGACTCTGACGGA |
| OsU6bT2 | gttgCATCACCAACACGCCGTTCC | aaacGGAACGGCGTGTTGGTGATG |

**Supplementary table 5. qPCR primer sequences used in study**

| Primer name | Forward primer (5’ to 3’) | Reverse primer (5’ to 3’) |
| --- | --- | --- |
| *ORF1* | CTGCCAGGTGCCTTCTATCC | TCCAGGCAAGGTTGTTGATGA |
| *ORF2* | GAAGGAAAAGCCTCCTCGCA | GGTCAAGTAGCTCGCATCGT |
| *ORF3* | AAGATCCAGCACTAACGCCC | TGTCCTCGAGGCTTCCATTG |
| *ORF5* | ATTCCCAAAGCATCATCCACGG | ATGTAGTTCCAGTCGGCACA |
| *ORF6* | CAGGCCATAATTGCTAAGATTGTCA | GTCTGGAGCAAATATCTTCAGGATT |
| *ORF7* | ACTTTCTGTGCTGGTGGC | TCCATGATCTCTATGTTGCCGT |
| *ORF8* | CCGAAAAGGAAAACCCTAGGCT | GTGGCTTTGGAGGAGAAGTG |
| *ORF9(Xa4)* | TTCATCCCTAACGGGAGCCT | TGATCTGATGATGGCCGTGC |
| *ORF10* | GCGTTCTTCGTCTCCCGTT | TCCGAATGTTGATGCTGCT |
| *ORF11* | CTGGACTTCAGAGCACTGGT | GGCGTGACACATTGCCATT |
| *ORF12* | CATGATCTCTGCGAGTATGCCG | CCACTGCCTTATGTTCAGTTCCC |
| *ORF13* | ATTGGTCAATGCTTATTGATTGCAC | GCTCCATGATCTCTGCGAGTATG |
| *OsActin* | TGGCATCTCTCAGCACATTCC | TGCACAATGGATGGGTCAGA |
| *XopA* | ATGAATTCTTTGAACACACAATTC | TTACTGCATCGATGCGCTGTCGCT |
| *OsPR1a* | CGTGTCGGCGTGGGTGT | GGCGAGTAGTTGCAGGTGATG |
| *OsPR1b* | TACGCCAGCCAGAGGAGC | GCCGAACCCCAGAAGAGG |
| *OsPR5* | CAACAGCAACTACCAAGTCGTCTT | CAAGGTGTCGTTTTATTCATCAACTTT |
| *OsPBZ1* | CACCATCTACACCATGAAGC | AGCACATCCGACTTTAGGAC |


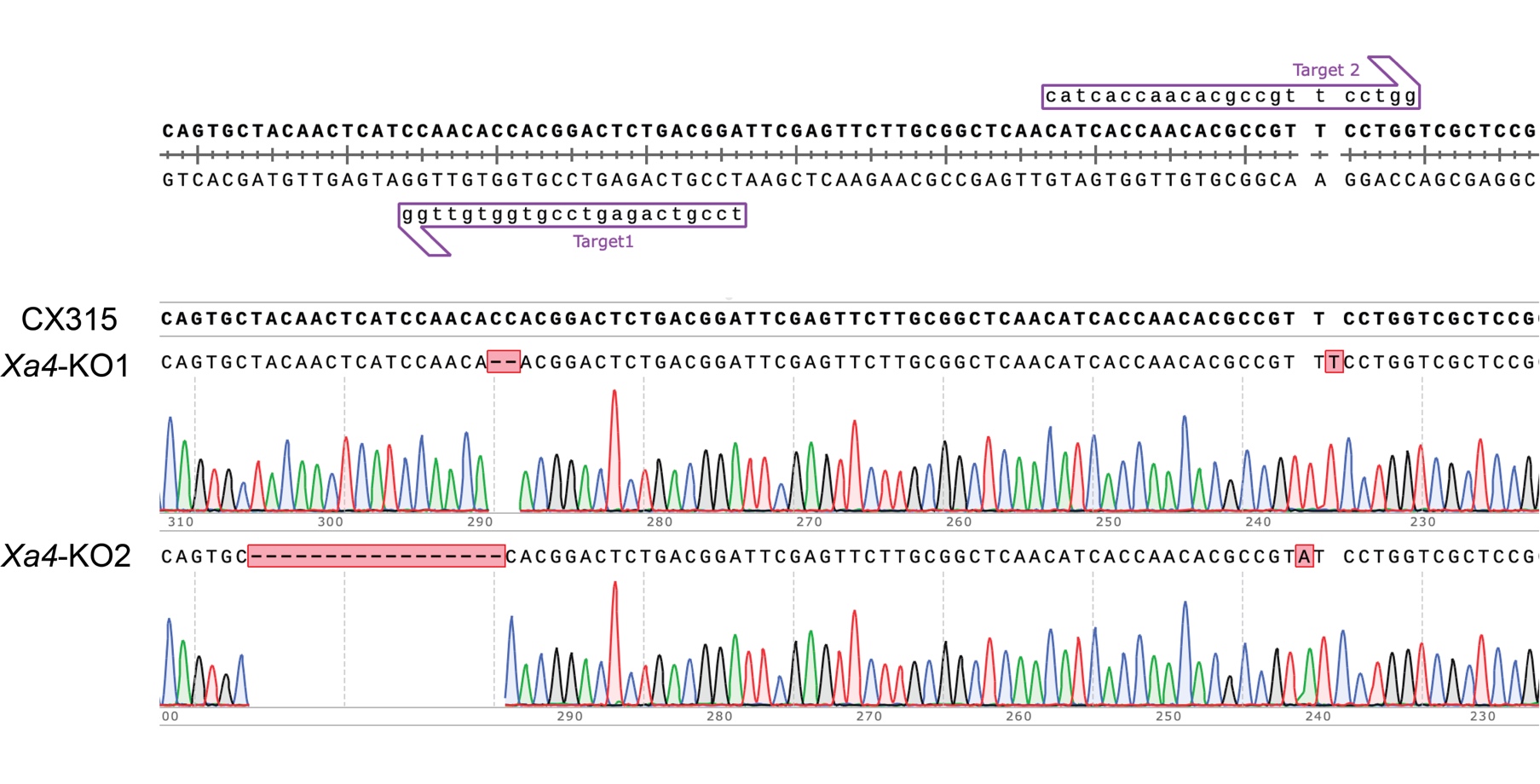


Supplementary figure S1: **Confirmation of Xa4 knockout in CX315 using CRISPR/Cas9 and sanger sequencing.** Target sites for CRISPR/Cas9-mediated editing of the Xa4 gene shown as primers. Two sgRNAs (Target 1 and Target 2) were designed to target exon 1 of Xa4. Sanger sequencing chromatograms of edited T_1_ plants showing indel mutations at the CRISPR target sites. Dash marks indicate the positions of base deletions and substitutions shown in boxes. The upper panels represent wild-type sequences from CX315, while the lower panels show mutations in knockout lines. These mutations led to frameshifts and premature stop codons or nonfunctional protein, confirming successful disruption of the Xa4 gene.


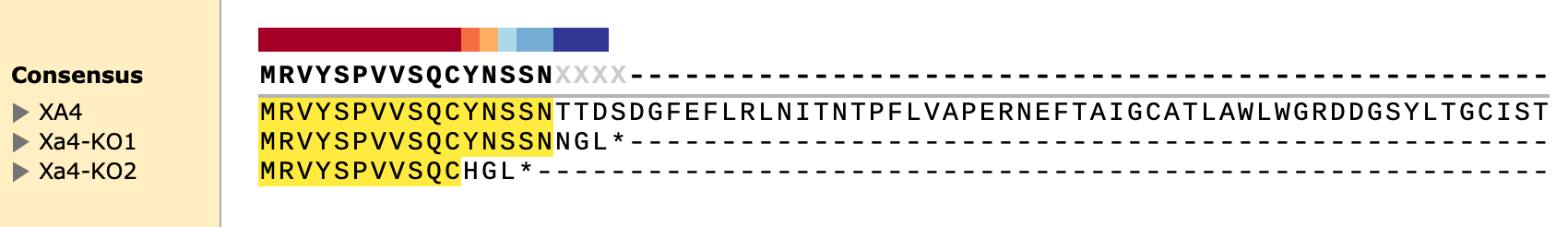


Supplementary figure S2: **Protein sequence alignment of Xa4 and its CRISPR/Cas9-edited knockout lines.** Amino acid alignment of wild-type Xa4 and two independent Xa4-KO lines (Xa4-KO1 and Xa4-KO2). The wild-type Xa4 encodes a full-length protein, whereas both knockout lines carry premature stop codons (indicated by asterisks) due to frameshift mutations, resulting in truncated proteins. The alignment demonstrates that the CRISPR-induced mutations disrupted the coding sequence early in the N-terminal region, abolishing the functional domain of the Xa4 protein.
